# Enhancing the Structural Stability and Diffusion Kinetics of a Tunnel-Phase Cathode by the Synergistic Effect of Cation-Anion Co-Doping for Advanced Sodium-Ion Batteries

**DOI:** 10.3390/molecules30112299

**Published:** 2025-05-23

**Authors:** Wenjing Shi, Xuezeng Duan, Zihan Xiao, Xiaofei Fan, Hao Zhang, Yan Wang, Lingyang Liu, Pengfang Zhang, Hengxiang Li

**Affiliations:** Shandong Provincial Key Laboratory of Chemical Energy Storage and Novel Cell Technology, School of Chemistry and Chemical Engineering, Liaocheng University, Liaocheng 252000, China; swjuser@163.com (W.S.); d1006443130@163.com (X.D.); 15725280930@163.com (Z.X.); 15169297589@163.com (X.F.); 13012987593@163.com (H.Z.); 13953420168@139.com (Y.W.); liulingy0425@163.com (L.L.); zhangpengfang111@163.com (P.Z.)

**Keywords:** sodium-ion batteries, cathode material, tunnel-phase Mn-based oxides, cation–anion co-doping, synergistic effect

## Abstract

Tunnel-structured Na_0.44_MnO_2_ (NMO) has been extensively studied as a potential cathode for sodium-ion batteries (SIBs) due to its favorable cycling endurance, cost-effectiveness, environmental benignity, and notable air-moisture stability. However, limitations, such as sluggish ion diffusion kinetics, an insufficient Na^+^ storage capacity, and an unsatisfactory Jahn–Teller effect, impede its widespread application. To address these problems, this study proposes a co-doping strategy that involves the simultaneous introduction of a cation and an anion. The optimized cathode Na_0.44_Mn_0.99_Ni_0.01_O_1.985_F_0.015_ demonstrates remarkable rate capabilities with average discharge capacities of 136.2, 133.0, 129.6, 124.0, 115.9, and 95.8 mAh g^−1^ under current rates ranging from 0.1 to 5 C. Furthermore, it also exhibits exceptional long-term cyclability, retaining 86.5% and 89.4% capacity retention at 1 and 5 C after 200 and 400 cycles, respectively, accompanied by nearly 100% Coulombic efficiency. A comprehensive structural characterization and experimental analysis reveal that the synergistic incorporation of Ni and F can effectively adjust the lattice parameters and alleviate the Jahn–Teller distortion of the NMO cathode, thereby resulting in enhanced structural integrity, rapid ion transfer dynamics, and excellent sodium storage performance. Consequently, this investigation establishes a significant approach for optimizing tunnel-phase Mn-based cathodes through the synergistic effect of cation and anion co-doping, which promotes the practical implementation of advanced SIBs.

## 1. Introduction

Owing to the natural abundance of Na resources, environmental friendliness, and work mechanism similar to lithium-ion batteries, sodium-ion batteries (SIBs) have attracted remarkable research attention as a promising candidate for large-scale smart grid and low-speed electric vehicle applications [1,2,3]. Cathode materials, as critical components, predominantly determine the capacity, lifespan, and energy density of SIBs [4,5]. Among the diverse cathodes, Mn-based transition metal oxides (Na_x_MnTO_2_, 0 < x ≤ 1, T = transition metal) have garnered widespread attention because of their high theoretical capacity, facile synthesis, and economic advantage [6,7]. These Na_x_MnTO_2_ compounds are typically divided into two categories: layer-structured oxides and tunnel-structured oxides. Layered cathodes, such as P2 and O3 phases, usually undergo an irreversible phase transition, transition metal dissolution, oxygen loss, and moisture sensitivity, resulting in severe structural degradation and pronounced volume fluctuation, ultimately causing serious capacity degradation and poor cycle stability [8,9,10]. In contrast, tunnel-structured oxides such as Na_0.44_MnO_2_ (NMO) have been widely investigated due to their sizeable S-shaped channels that can accommodate Na^+^ insertion and extraction with lower lattice strain and volume variations, demonstrating decent cycle life [11,12,13]. Nevertheless, sluggish ion diffusion kinetics, an insufficient Na^+^ storage capacity, and an unsatisfactory Jahn–Teller effect significantly restrict its practical application [14,15].

To address the abovementioned challenges for NMO, various modification strategies have been explored, including surface coating [16,17], ion doping [18,19], and composite structure construction [20,21]. Ion doping, in particular, is recognized as an efficient approach to strengthen structural integrity and enhance sodium storage capabilities. For instance, the introduction of low-valence cations, such as Li^+^ [22,23], Mg^2+^ [24], Co^3+^ [25], B^3+^ [26], Fe^3+^ [27], and Ti^4+^ [28,29], into NMO can effectively stabilize the crystal structure by inhibiting the J–T distortion, decrease the (de)intercalation energy barrier of Na^+^, and accelerate electrode kinetics, resulting in an improvement in the rate performance and cycle stability. In addition, recent investigations have reported the influence of doping NMO with high-valence cations. Introducing Nb^5+^ into the tunnel structure, for example, reinforces structural robustness and promotes Na^+^ diffusion kinetics, as well as increases electronic conductivity, leading to outstanding electrochemical performance [30]. Introducing a trace amount of W^6+^ [31] or Mo^6+^ [32] can induce the tunnel structure to transform into a layered structure, and the optimized material exhibits a higher specific capacity, extraordinary energy density, and excellent air stability. In addition to cation doping, the effects of anion F-doping on Na_x_MnTO_2_ have also been extensively explored [33,34,35]. Research indicates that F-substitution is beneficial to enhancing the redox potential, improving ionic conductivity, and preventing transition metal ion migration [36].

Compared with the single-site substitution technique, dual-site co-substitution provides a significantly synergistic effect on the adjustment of the crystal and electronic structures of Na_x_MnTO_2_ cathodes, which can efficiently inhibit structural destruction caused by the J–T effect, suppress Mn^2+^ dissolution, and facilitate charge transfer kinetics, thereby resulting in superior electrochemical properties. Na_0.44_Cu_x_Mn_1−x_O_1.93_F_0.07_ (x = 0, 0.02, 0.05, 0.1, and 0.2) materials featuring Cu/F co-doping have been designed. The results show that an appropriate amount of Cu^2+^ and F^−^ doping still preserves the tunnel structure. Cu^2+^ introduction can restrain the complex phase transition process, thus leading to smooth charge/discharge curves and decent cycling stability, while F-introduction boosts ion diffusion kinetics, resulting in enhanced rate performance [37]. A Na_0.44_Mn_0.895_Ti_0.1_Mg_0.005_O_2_ (NMO-TM) cathode with Ti/Mg co-doping has been reported. Ti/Mg co-doping enlarged the Na^+^ transport channel, reduced the morphology size, and diminished the ion diffusion distance. The synergistic action of Ti and Mg endowed the NMO-TM cathode with a relatively stable structure, resulting in a higher capacity at a high rate (80 mAh g^−1^ at 20 C) [14]. Moreover, a Zn/F co-doped P2-Na_0.67_Zn_0.05_Ni_0.15_Fe_0.20_Mn_0.60_O_1.95_F_0.05_ material was synthesized via the co-precipitation method. The experimental results and theoretical calculation analysis indicated that Zn/F co-substitution modulated the localized electron configuration, reinforced oxidation–reduction kinetics, and alleviated structural distortion in neighboring transition metal coordination units, thereby exhibiting improved overall electrochemical behavior [10]. In addition, Mg/F [5], Li/F [38], and Al/F [39] dual-ion co-doping in Na_x_MnTO_2_ materials have also been extensively reported, which demonstrated that the synergistic impact of cationic and anionic dopants could effectively utilize their complementary benefits and ultimately achieve cathodes with enhanced sodium storage performance.

Inspired by the previously reported studies, dual-site co-doping strategies are believed to hold considerable potential for improving the structural integrity and electrochemical properties of tunnel-type cathodes. In this work, we propose a Ni/F co-doping approach to preserve the tunnel structure and optimize the comprehensive electrochemical properties of the NMO material. The results demonstrate that Ni-introduction improved cycle stability by inhibiting Na^+^/vacancy and charge ordering, while F-introduction enhanced the specific capacity by appropriately increasing the concentration of active manganese ions. Consequently, the Ni/F co-substitution could efficiently reduce the impact of the J–T distortion, enhance structural stability, and accelerate the ion transport kinetics of the NMO material. As a result, the optimized cathode Na_0.44_Mn_0.99_Ni_0.01_O_1.985_F_0.015_ (NMONi1F1.5) displayed superb rate performance (95.8 mAh g^−1^ at 5 C) and remarkable long-term cycling stability (89.4% capacity retention at 5 C after 400 cycles). Furthermore, ex-situ X-ray diffraction (XRD) analysis further confirmed the structural reversibility of NMONi1F1.5 during Na^+^ insertion/extraction processes. This strategy of cooperative interaction between cation and anion doping provides a pathway for developing high-performance Mn-based cathodes for SIBs.

## 2. Results and Discussion

**Figure 1:**
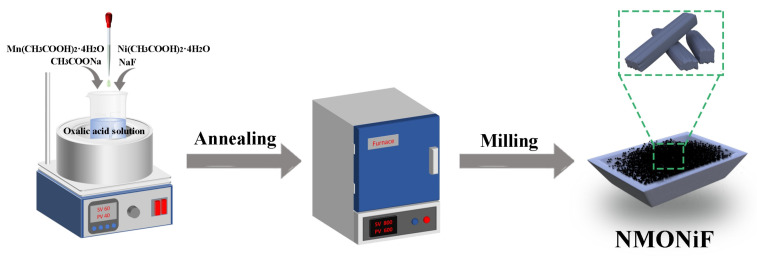
The schematic diagram for the fabrication of NMONiF.

A series of tunnel-type materials featuring Ni-substitution (Na_0.44_Mn_1−x_Ni_x_O_2_, x = 0.005, 0.01, 0.015, 0.02, named as NMONi0.5, NMONi1, NMONi1.5, NMONi2), F-substitution (Na_0.44_MnO_2−x_F_x_, x = 0.005, 0.01, 0.015, 0.02, named as NMOF0.5, NMOF1, NMOF1.5, NMOF2), and Ni/F co-substitution (Na_0.44_Mn_0.99_Ni_0.01_O_2−x_F_x_, x = 0.005, 0.01, 0.015, 0.02, named as NMONi1F0.5, NMONi1F1, NMONi1F1.5, NMONi1F2) were synthesized using a co-precipitation method, as depicted in Figure 1. A thermogravimetric (TG) analysis was performed to evaluate the mass retention of the NMO, NMOF1.5, NMONi1, and NMONi1F1.5 samples (Figure S1). The mass loss observed across all the samples predominantly occurred in two distinct stages: the first stage (120 to 160 °C) corresponded to the decomposition of crystalline water; the second stage (250 to 340 °C) was attributed to the thermal decomposition of oxalic acid, leading to the formation of CO_2_ and intermediate metal oxalate compounds [40]. As the heat treatment temperature further increased, the samples exhibited negligible mass loss, demonstrating effective thermal stabilization with a final mass retention rate of approximately 47.5%.

**Figure 2:**
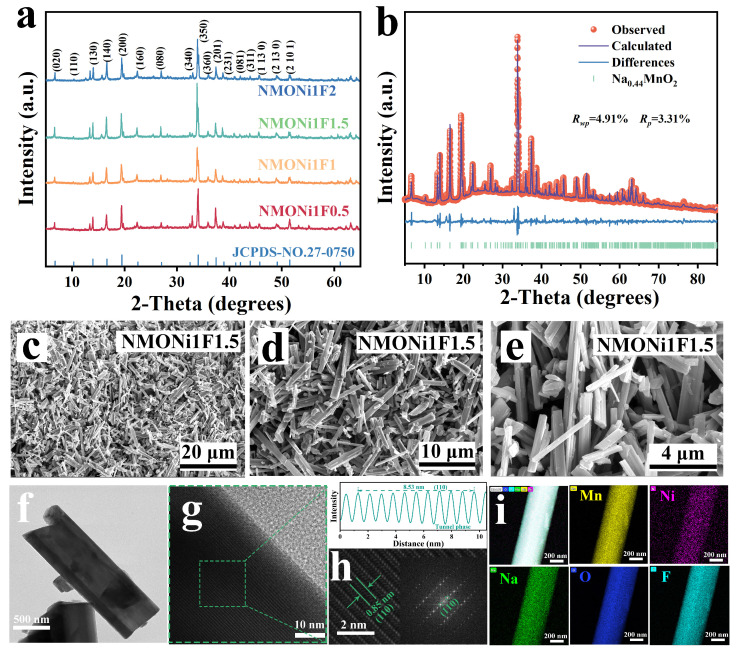
Structural and morphological analysis: (**a**) XRD pattern of NMONiF; (**b**) Rietveld refinement of NMONi1F1.5; (**c**–**e**) SEM and TEM images of NMONi1F1.5; (**f**–**i**) TEM, HRTEM (with the corresponding FFT patterns and line-scanning intensity profiles), and EDS mapping images of NMONi1F1.5.

The crystal structures of the NMONiF, NMONi, and NMOF electrodes, as determined by XRD analysis, are presented in Figure 2a and Figure S2, where the diffraction peaks are well consistent with the orthorhombic tunnel phase, exhibiting the *Pbam* space group (JCPDS no. 27–0750). When the Ni content reached 2%, the distinct diffraction peaks appearred around 2θ = 33° in NMONi2, demonstrating the existence of an Mn_2_O_3_ impurity (Figure S2a). However, the XRD spectrum of NMONiF (Figure 2a) and NMOF (Figure S2b) still maintained the characteristic diffraction peaks of the tunnel phase, suggesting that the trace F^−^ ion doping effectively preserved the crystallinity of the tunnel phase without the generation of impurity phases. The precise cell parameters of NMONi1F1.5 were determined by the XRD Rietveld refinement, as illustrated in Figure 2b. The lattice parameters *a*, *b*, and *c* of NMONi1F1.5 were increased to 9.091, 26.462, and 2.828 Å compared to those of NMO (*a* = 9.081, *b* = 26.451, and *c* = 2.823 Å), as listed in Table S1. Moreover, the unit cell volume of NMONi1F1.5 increased from 678.073 Å^3^ (NMO) to 680.430 Å^3^. These changes, namely the expansion of the unit cell volume and the increase in lattice parameters, are beneficial to enhance Na^+^ diffusion behavior during the charging and discharging processes [30,41].

Scanning electron microscopy (SEM), transmission electron microscopy (TEM), and high-resolution TEM (HRTEM) were employed to investigate the crystal structure and morphology characteristics of the synthesized NMONi, NMOF, and NMONiF samples. As depicted in Figure 2c–f and Figures S3–S5, the synthesized cathodes exhibited even rod-like morphology with a smooth surface and better particle uniformity, contributing to improved electrochemical stability. In addition, no significant differences in morphology were observed among the samples, indicating that an appropriate amount of Ni doping, F doping, or Ni/F co-doping did not affect the structure and morphology of the tunnel type. Moreover, the energy dispersive X-ray spectroscopy (EDS) elemental mapping images revealed a uniform distribution of Na, Mn, O, Ni, or F elements arcoss the NMONi1 and NMOF1.5 samples (Figures S3 and S4). The HRTEM images and corresponding Fast Fourier Transform (FFT) patterns of NMONi1F1.5 are displayed in Figure 2g,h. The clear diffraction spots in the FFT images confirmed the high crystallinity of the tunnel structure. A further analysis of the line-scan intensity profiles (Figure 2h) demonstrated that the interplanar spacing between neighboring lattice fringes was approximately 0.85 nm, corresponding to the (110) crystal planes of the tunnel phase. The EDS elemental mapping images, as shown in Figure 2i, verified the homogeneous distribution of all elements throughout the NMONi1F1.5 sample, demonstrating the successful introduction of both Ni and F^−^ ions into the host structure.

**Figure 3:**
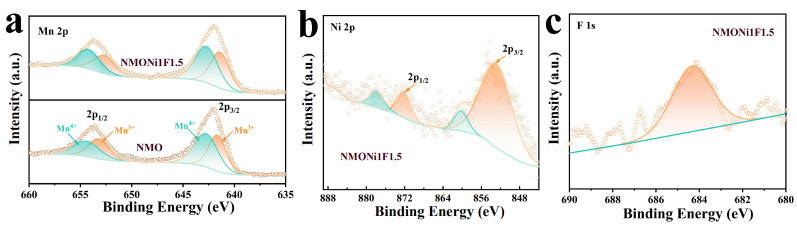
(**a**) High-resolution Mn 2p XPS spectra for NMO and NMONi1F1.5; (**b**,**c**) high-resolution Ni 2p and F 1s XPS spectra for NMONi1F1.5.

The X-ray photoelectron spectroscopy (XPS) spectra of the NMO and NMONi1F1.5 samples are displayed in Figure 3, which offers a comprehensive evaluation of the elemental compositions and valence states of the cathodes. The high-resolution Mn 2p spectra (Figure 3a) displayed two distinct peaks for Mn 2p_1/2_ and Mn 2p_3/2_ positioned at approximately 653.6 and 642.2 eV, respectively. These peaks were deconvoluted into Mn^3+^ (641.4 and 652.7 eV) and Mn^4+^ (642.7 and 654.3 eV), confirming the coexistence of multiple oxidation states [42,43]. Notably, the semi-quantitative ratio of Mn^4+^/Mn^3+^ in NMONi1F1.5 was slightly higher than that of NMO. This phenomenon could be attributed to the substitution of some Mn^3+^ ions by Ni^2+^ ions, which would elevate the average oxidation state of Mn and suppress the J–T distortion [44,45], thus enhancing the structural stability of the NMONi1F1.5 cathode. The Ni 2p spectra, as depicted in Figure 3b, exhibited the characteristic peaks related to Ni 2p_3/2_ and Ni 2p_1/2_. The central positions of these peaks were located at approximately 854.7 and 872.7 eV [46,47], respectively. Figure 3c shows the high-resolution F 1s spectra around 684.5 eV for NMONi1F1.5, indicating the presence of F^−^ ions in the material [37,39]. The high-resolution Na 1s spectrum was located at 1070.8 eV (Figure S6a), indicating the Na^+^ species [48]. The O 1s spectrum exhibited three distinct peaks (Figure S6b). The peak at 529.7 eV corresponded to lattice oxygen, while the peaks observed at 531.4 and 533.9 eV were associated with surface-adsorbed oxygen species [49,50]. Moreover, based on the XPS combined with the TEM mapping analysis, the atomic percentages of Na, Mn, Ni, F, and O in NMONi1F1.5 were 11.85%, 27.51%, 0.32%, 59.59%, and 0.73%, which closely matched the theoretical stoichiometric ratio of 12.79%, 28.78%, 0.29%, 57.70%, and 0.44%.

To explore the influence of the Ni or F doping ratio on the electrochemical performance of NMO, a cycling performance assessment was conducted at 1 C on both NMONi and NMOF materials, as illustrated in Figures S7 and S8. These results revealed that the NMONi1 and NMOF1.5 samples maintained a relatively high discharge capacity after 200 cycles. Consequently, doping with 1% Ni and 1.5% F were determined as the optimal dopant concentrations. As demonstrated in Figure S9a, the single-site doped cathodes of NMONi1 and NNOF1.5 delivered reversible capacities of 107.1 and 119.9 mAh g^−1^ at 0.1 C for the second cycle. Figure S9b exhibits the rate performance, where the average discharge capacities of NMONi1 and NMOF1.5 were 106.6−93.2 mAh g^−1^ and 119.3−95.3 mAh g^−1^, respectively, across current rates from 0.1 to 5 C. These findings imply that an appropriate amount of F-introduction has the potential to improve the specific capacity of the tunnel-type cathode. This improvement is attributed to the fact that the introduction of F^−^ leads to a slight increase in the proportion of Mn^3+^ ions for charge compensation [37]. In addition, the cycling performances of the NMONi1 and NMOF1.5 cathodes at 5 C are presented in Figure S9c,d. It was found that NMONi1 maintained decent cycle stability with 79.9% capacity retention after 400 cycles compared with that of NMOF1.5 (75.4%), revealing that Ni introduction was conducive to enhancing the cycling lifespan of the tunnel-type cathode by inhibiting Na^+^/vacancy and charge ordering.

**Figure 4:**
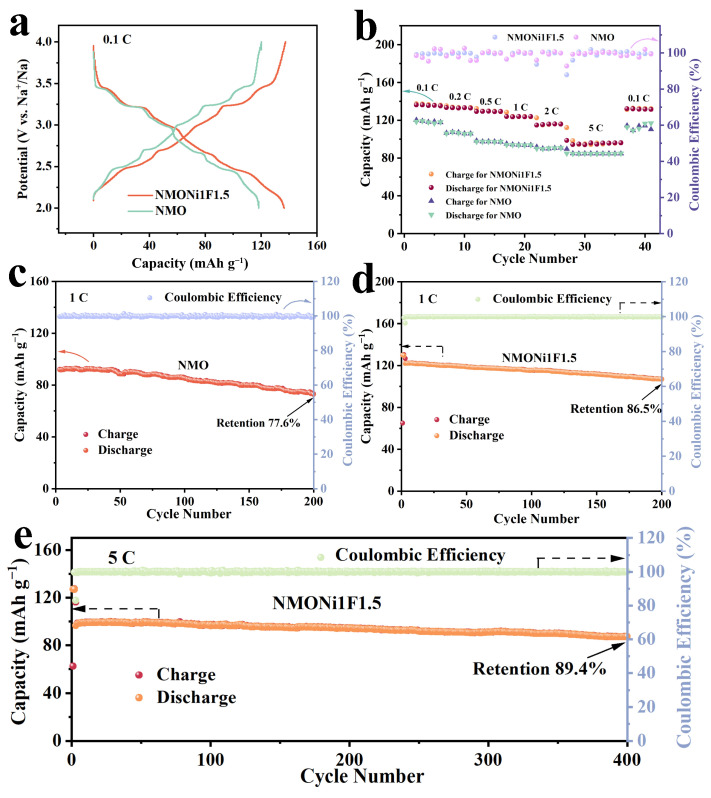
Electrochemical performance of NMO and NMONi1F1.5 cathodes: (**a**) GCD profiles for the second cycle at 0.1 C; (**b**) rate capability; (**c**–**e**) long-term cycling performance at 1 and 5 C.

Figure 4a illustrates the galvanostatic charge–discharge (GCD) curves of un-doped NMO and Ni/F dual-site co-doped NMONi1F1.5 at 0.1 C for the second cycle. Notably, the NMONi1F1.5 sample exhibited a superior discharge capacity of 136.5 mAh g^−1^, exceeding the 118.3 mAh g^−1^ observed for the NMO cathode. Furthermore, as depicted in Figure 4b, the NMONi1F1.5 cathode also displayed outstanding rate capabilities, delivering average discharge capacities of 136.2, 133.0, 129.6, 124.0, 115.9, and 95.8 mAh g^−1^ at rates of 0.1, 0.2, 0.5, 1, 2, and 5 C, respectively, which was much higher than those of NMO. Impressively, the average discharge capacity of the NMONi1F1.5 cathode remained 131.7 mAh g^−1^ when the current rate returned to 0.1 C, revealing exceptional structural reversibility. The cycling tests of the NMO and NMONiF cathodes at 1 C are presented in Figure 4c,d and Figure S10. The results demonstrate that the NMONi1F1.5 cathode exhibited an excellent capacity retention ratio of 86.5% after 200 cycles, which was much better than NMO (77.6% capacity retention), NMONi1F0.5 (84.8% capacity retention), NMONi1F1 (83.9% capacity retention), and NMONi1F2 (72.4% capacity retention). More significantly, as illustrated in Figure 4e, NMONi1F1.5 displayed an excellent capacity retention of 89.4% at a high current rate of 5 C after 400 cycles, suggesting superb structural stability. As summarized in Table S2, the NMONi1F1.5 cathode also exhibited competitive rate property and cycling durability compared with the other reported Mn-based tunnel-type electrodes. These results conclusively confirm that co-doping with Ni and F is an effective strategy to enhance both charge storage capacity and long-term cycling durability.

**Figure 5:**
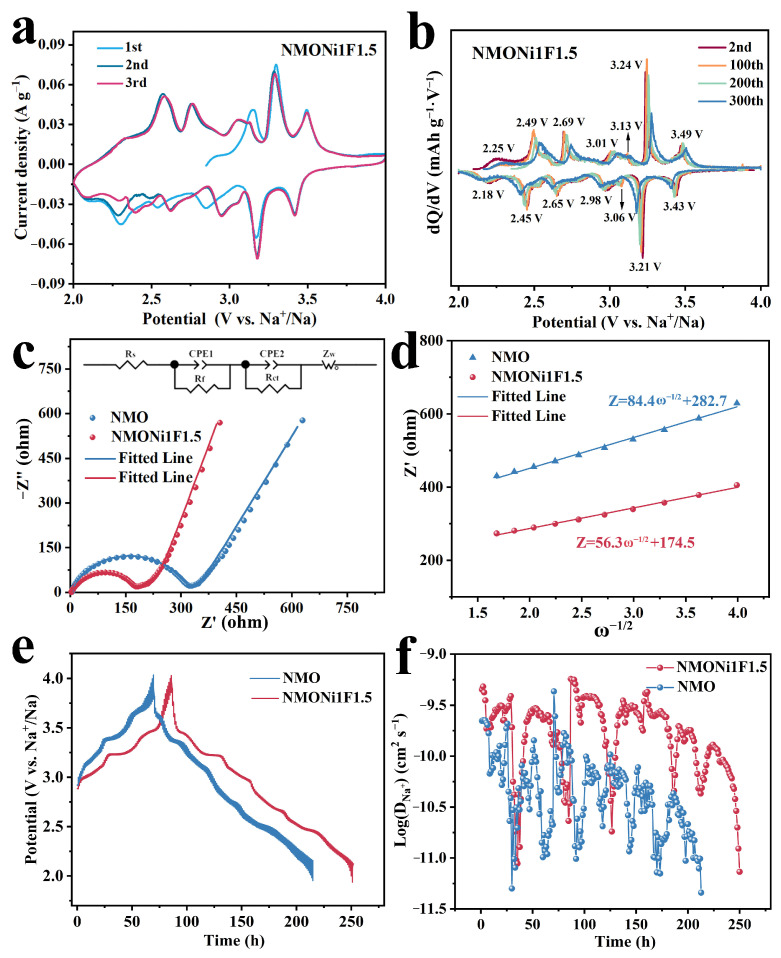
Electrochemical performance of the electrode: (**a**) the CV curves of NMONi1F1.5 at 0.1 mV s^−1^; (**b**) dQ/dV profiles for NMONi1F1.5 (2.0−4.0 V); (**c**) EIS comparison plots of NMO and NMONi1F1.5 (with equivalent circuit diagram inset); (**d**) the relationship between the Z’ and ω^−1/2^ for NMO and NMONi1F1.5; (**e**) GITT measurements of NMO and NMONi1F1.5; (**f**) comparison of the calculated Na^+^ diffusion coefficients for NMO and NMONi1F1.5.

As depicted in Figure 5a,b, the electrochemical characteristics of the NMONi1F1.5 sample were examined by cyclic voltammetry (CV) and a dQ/dV plot at 0.1 mV s^−1^ under various cycles. The dQ/dV profiles demonstrate seven pairs of reversible redox peaks for NMONi1F1.5, centered at approximately 3.49/3.43, 3.24/3.21, 3.13/3.06, 3.01/2.98, 2.69/2.65, 2.49/2.45, and 2.25/2.18 V with the potential ranging from 2.0 to 4.0 V, corresponding to a series of phase transition processes. The electrochemical impedance spectroscopy (EIS) measurement results are presented in Figure 5c,d, which were acquired to evaluate the interfacial charge transfer kinetics for NMO and NMONi1F1.5. Nyquist plots were fitted using an equivalent circuit diagram, where the *R*_s_, *R*_f_, *R*_ct_, and σ represent ohmic resistance, electrode/electrolyte interface resistance, charge transfer resistance, and Warburg’s coefficient (Figure 5c), respectively. As a result, the EIS spectra obtained for the NMONi1F1.5 electrode presented smaller values of *R*_s_ (3.43 Ω), *R*_f_ (35.8 Ω), *R*_ct_ (155.8 Ω), and σ (56.3) compared with those of NMO (5.5 Ω, 48.5 Ω, 240.2 Ω, and 84.4). To further assess the Na^+^ diffusion kinetics in the NMO and NMONi1F1.5 cathodes, the galvanostatic intermittent titration technique (GITT) tests were performed (Figure 5e,f and Figure S11). NMONi1F1.5 maintained an average Na^+^ diffusion coefficient of 2.1 × 10^−10^ cm^2^ s^−1^, significantly exceeding the value obtained for NMO (5.7 × 10^−11^ cm^2^ s^−1^). These results confirm that the Ni and F co-introduction strategy boosted Na^+^ diffusion kinetics. Therefore, the comprehensive electrochemical evaluation substantiates the effectiveness of the dual-ion modification strategy in enhancing Na^+^ storage and diffusion kinetic performances of the tunnel-phase cathode.

**Figure 6:**
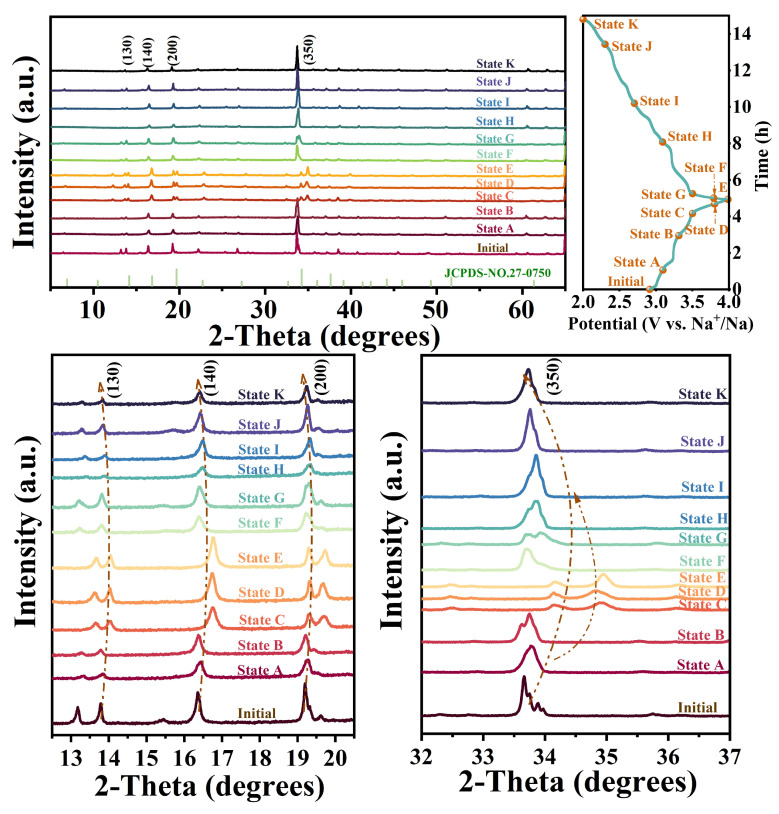
Ex-situ XRD of NMONi1F1.5 collected at 0.1 C for the first charging/discharging process.

To further elucidate the underlying energy storage mechanism, ex-situ XRD was conducted to study the structural evolution of the NMONi1F1.5 cathode during the Na^+^ insertion and extraction processes. As illustrated in Figure 6, some diffraction peaks, including (130), (140), and (350), progressively shifted towards higher angles during the charging process, revealing the lattice parameter contraction of the NMONi1F1.5 cathode. Notably, the (350) diffraction peak split and generated a new distinct reflection (0 10 0) between state C and state E. This structural change suggests the lattice shrinkage arose from the decreased electrostatic repulsion force of O−O during the desodiation process [30]. During the subsequent discharging process, these characteristic peaks gradually returned to their original positions, and the newly generated (0 10 0) peak merged back into the (350) peak as the potential transitioned from state E to state K. Therefore, these diffraction peaks position variations confirm the superior structural reversibility associated with Na^+^ extraction/insertion processes.

## 3. Experimental Section

### 3.1. Materials Preparation

Following a standard procedure, stoichiometric ratios of CH_3_COONa (AR, 99%), Mn(CH_3_COO)_2_·4H_2_O (AR, 99%), Ni(CH_3_COO)_2_·4H_2_O (AR, 99%), oxalic acid (AR, 99%), and NaF (AR, 99%) were homogenized in deionized water under continuous stirring at 60 °C for 6 h, followed by dehydration in an oven at 80 °C for 12 h. The resultant intermediate products subsequently underwent calcination at 900 °C for 12 h under an air atmosphere. Finally, the resulting cathodes Na_0.44_Mn_0.99_Ni_0.01_O_1.995_F_0.005_, Na_0.44_Mn_0.99_Ni_0.01_O_1.99_F_0.01_, Na_0.44_Mn_0.99_Ni_0.01_O_1.985_F_0.015_, and Na_0.44_Mn_0.99_Ni_0.01_O_1.98_F_0.02_ were designated as NMONi1F0.5, NMONi1F1, NMONi1F1.5, and NMONi1F2, respectively. Additionally, a series of Ni and F single-doped tunnel-phase cathodes with various Ni/Mn or F/O molar ratios of 0.005:0.995, 0.01:0.99, 0.015:0.985, and 0.02:0.98 were synthesized, which were identified as NMONi0.5, NMONi1, NMONi1.5, NMONi2, NMOF0.5, NMOF1, NMOF1.5, and NMOF2, respectively. For comparison, the tunnel-phase NMO cathode was prepared using a similar method but without Ni(CH_3_COO)_2_·4H_2_O and NaF, which was described in our previous work [22]. All relevant reagents were purchased from Sinopharm Chemical Reagent Co., Ltd. (Shanghai, China) and used without any purification.

### 3.2. Materials Characterization

XRD patterns of samples were recorded on a Rigaku SmartLab 9 kW instrument (Cu Kα radiation, λ = 0.15406 nm; Tokyo, Japan) within a 2θ range of 5° to 65°. Sample morphology was characterized via SEM (GX4, Bainbridge, GA, USA) and TEM (F200X, Talos, Houston, TX, USA). Elemental composition and corresponding elemental mapping distributions were determined using EDS spectra. Chemical bonding state and composition of the samples were investigated via XPS (Escalab Xi+, Pittsburgh, PA, USA). TG analysis of the oxalate precursor was conducted on a thermal analyzer (Discovery 550, Gaithersburg, MD, USA) with a scan rate of 10 °C min^−1^ in the temperature range from 50 to 950 °C under an oxygen atmosphere.

### 3.3. Electrochemical Measurements

The homogeneous slurry was prepared by combining 80 wt% cathode material, 10 wt% Super P, and 10 wt% polyvinylidene fluoride within N-methyl-pyrrolidinone solvent. This mixture was uniformly deposited onto Al foil current collectors, followed by drying at 100 °C for 12 h in a vacuum oven. CR2032 coin cells were assembled using NMO, NMONi, NMOF, or NMONiF as working electrode, 1.0 M NaClO_4_ dissolved in ethylene carbonate and propylene carbonate (1:1 in volume) with 2 wt% fluoroethylene carbonate as the electrolyte, metallic sodium as a counter electrode, and glass fiber as the separator. CV and EIS measurements were carried out using a CHI660E (Chenhua, Shanghai, China) electrochemical workstation. The GCD, cycling stability, rate performance, and GITT were evaluated using a LAND CT3002A testing system (Wuhan, China).

## 4. Conclusions

In summary, a cation–anion dual-site doping strategy has been employed to modify the NMO cathode through the incorporation of Ni^2+^ and F^−^ ions. This method can preserve the structural integrity of the tunnel-phase framework. As a result, the introduction of Ni^2+^ and F^−^ ions into the tunnel structure supports a favorable structural regulation, resulting in improved structural stability, energy storage capacity, and Na^+^ diffusion kinetics. The electrochemical evaluations revealed that the Ni-doped cathode (NMONi1) maintained a 79.9% capacity retention at 5 C after 400 cycles, demonstrating an excellent long-term cycling stability. However, the F-doped cathode (NMOF1.5) exhibited a better discharge capacity of 119.3 and 95.3 mAh g^−1^ at 0.1 and 5 C, and it achieved a decent cycling stability with a 75.4% capacity retention after 400 cycles at 5 C. Importantly, due to the synergistic interaction between Ni^2+^ and F^−^, the optimized NMONi1F1.5 cathode delivered exceptional long-term cycling performance with 86.5% and 89.4% capacity retention at 1 and 5 C after 200 and 400 cycles, coupled with outstanding rate performance for average discharge capacities of 136.2, 133.0, 129.6, 124.0, 115.9, and 95.8 mAh g^−1^ at 0.1, 0.2, 0.5, 1.0, 2.0, and 5 C, respectively. This study provides strategic insights for the design of advanced transition metal oxide cathodes with superior cycling durability, high storage capacity, and rapid Na^+^ diffusion kinetics, thereby promoting further practical implementation in SIBs.

## Data Availability

Data will be made available on request.

## Supplementary Materials

The following supporting information can be downloaded at: https://www.mdpi.com/article/10.3390/molecules30112299/s1. Figure S1: TG analysis of oxalate precursor for the NMO, NMOF1.5, NMONi1, and NMONi1F1.5 samples; Figure S2: The XRD patterns of (**a**) NMONi and (**b**) NMOF cathodes; Figure S3: SEM and corresponding EDS mapping images of NMONi cathodes; Figure S4: SEM and corresponding EDS mapping images of NMOF cathodes; Figure S5: SEM images of NMONiF cathodes; Figure S6: High-resolution Na 1s and O 1s XPS spectra for NMONi1F1.5 sample; Figure S7: Cycling performance of the NMONi cathodes at 1 C; Figure S8: Cycling performance of the NMOF cathodes at 1 C; Figure S9: Electrochemical performance of NMONi1 and NMOF1.5 cathodes. (**a**) The galvanostatic charge–discharge curves for the second cycle at 0.1 C, (**b**) rate capability, (**c**,**d**) cycling performance at 5 C; Figure S10: Cycling performance of the NMONiF cathodes at 1 C; Figure S11: (**a**,**b**) Schematic diagram of a single-step titration of NMO and NMONi1F1.5 during GITT measurement. The linear relationship of (**c**) NMO and (**d**) NMONi1F1.5 between the potential and τ^1/2^ during the titration; Table S1: Summary of fitted parameters in XRD Rietveld refinement [22]; Table S2: The comparison of electrochemical performance between the previously reported Mn-based tunnel-phase cathodes and this work [24,25,27,30,32,37,51,52,53,54].
