# Supplementary material for: Enhancing the Structural Stability and Diffusion Kinetics of a Tunnel-Phase Cathode by the Synergistic Effect of Cation-Anion Co-Doping for Advanced Sodium-Ion Batteries
Source: Molecules. 2025 May 23;30(11):2299. doi: 10.3390/molecules30112299 (PMC12156011; doi:10.3390/molecules30112299)
Supplement: Supplementary file 1 [file molecules-30-02299-s001.zip › molecules-3643767-supplementary.pdf]

Supplementary Material

Enhancing the Structural Stability and Diffusion Kinetics of a Tunnel-Phase Cathode by the Synergistic Effect of Cation-Anion Co-Doping for Advanced Sodium-Ion Batteries

Wenjing Shi, Xuezeng Duan, Zihan Xiao, Xiaofei Fan, Hao Zhang, Yan Wang, Lingyang Liu, Pengfang Zhang and Hengxiang Li*

Shandong Provincial Key Laboratory of Chemical Energy Storage and Novel Cell Technology, School of Chemistry and Chemical Engineering, Liaocheng University, Liaocheng, 252000, China

* Correspondence: lihengxiangg@163.com (H.L.)

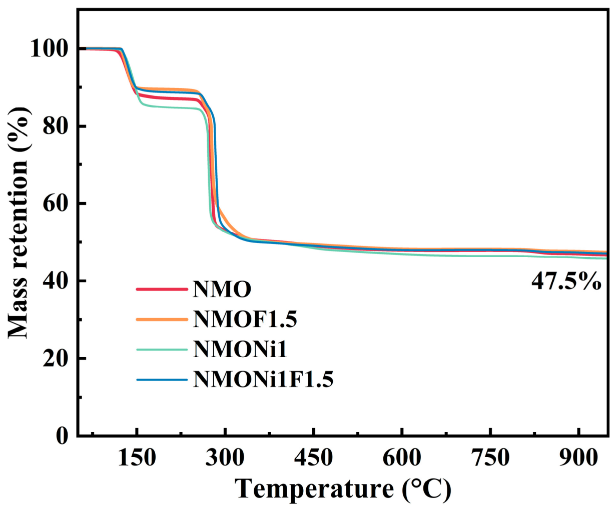

Figure S1. TG analysis of oxalate precursor for the NMO, NMOF1.5, NMONi1, and NMONi1F1.5 samples.

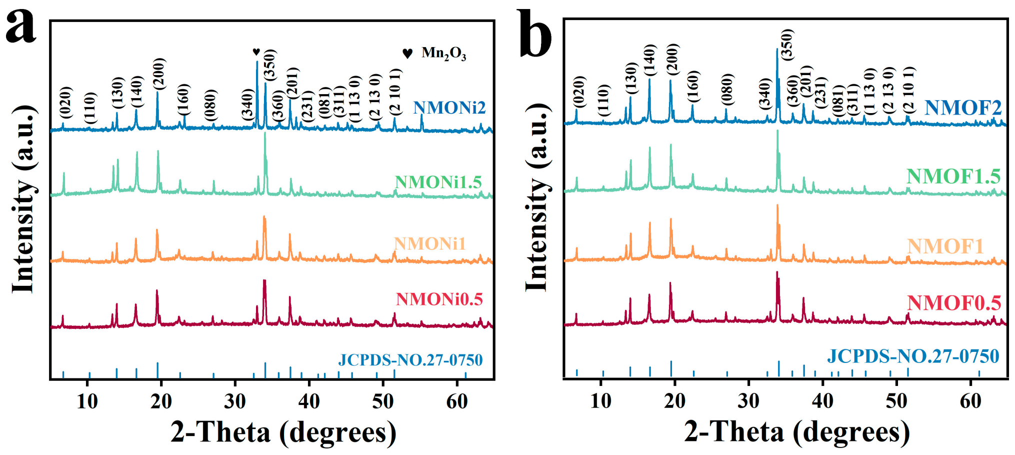

Figure S2. The XRD patterns of (a) NMONi and (b) NMOF cathodes.

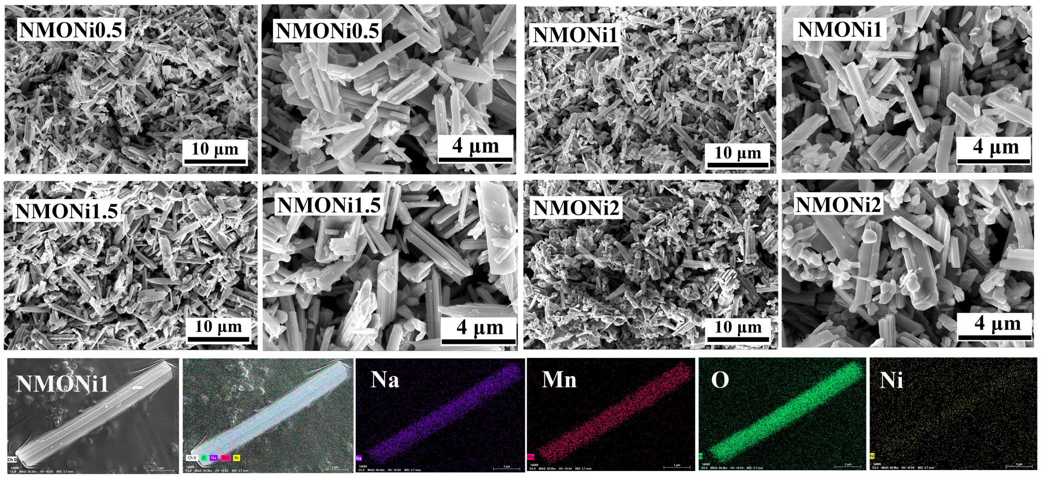

Figure S3. SEM and corresponding EDS mapping images of NMONi cathodes.

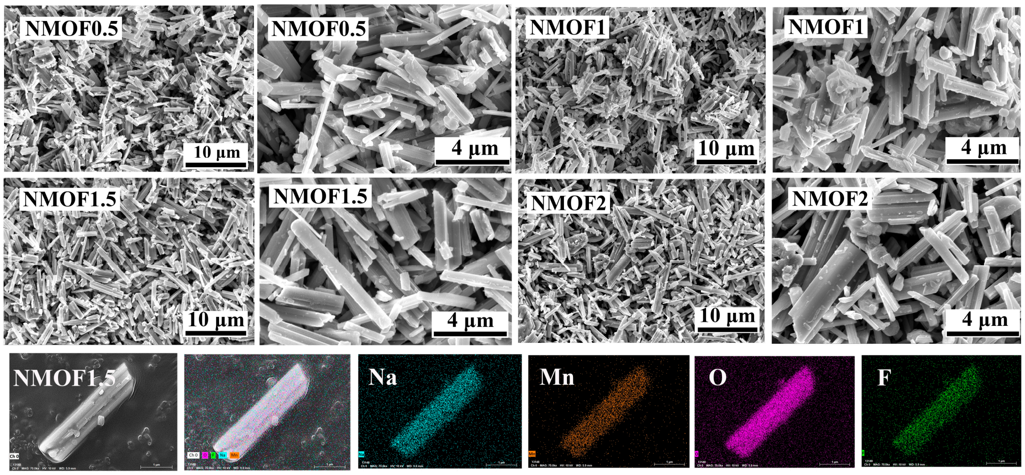

Figure S4. SEM and corresponding EDS mapping images of NMOF cathodes.

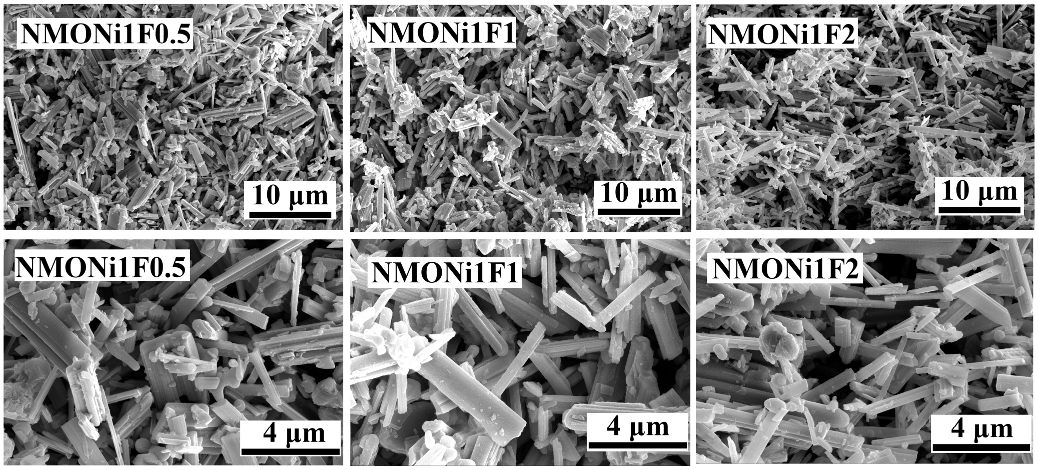

Figure S5. SEM images of NMONiF cathodes.

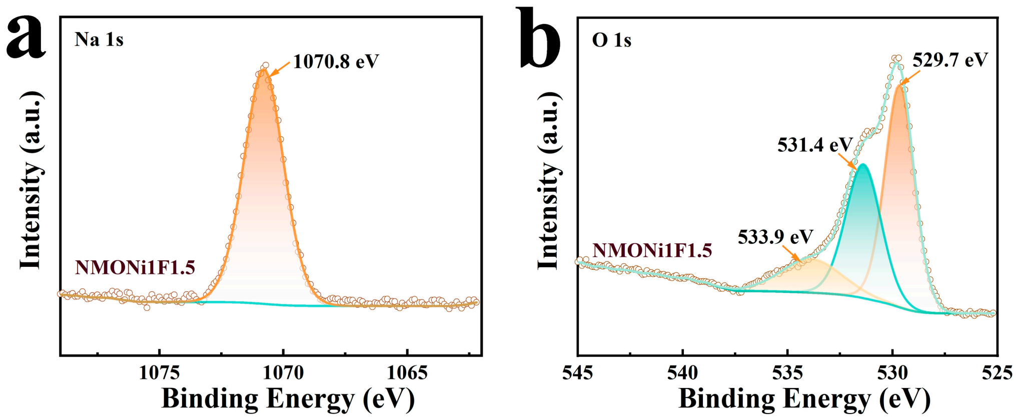

Figure S6. High-resolution Na 1s (a) and O 1s (b) XPS spectra for the NMONi1F1.5 sample.

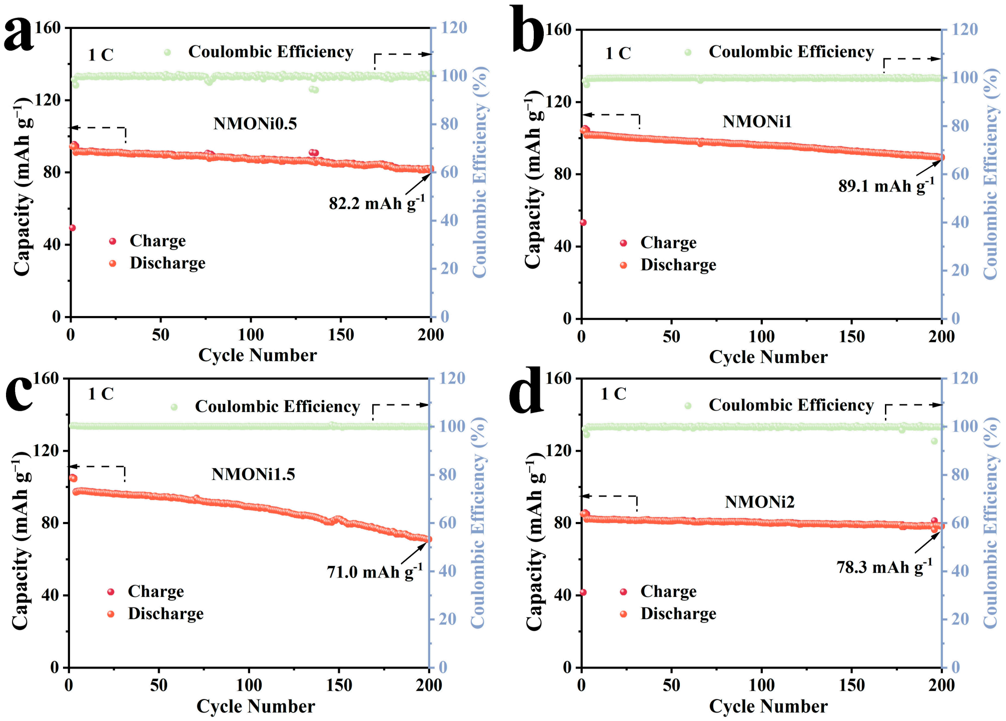

Figure S7. Cycling performance of the NMONi cathodes at 1 C: (a) NMONi0.5, (b) NMONi1, (c) NMONi1.5, (d) NMONi2.

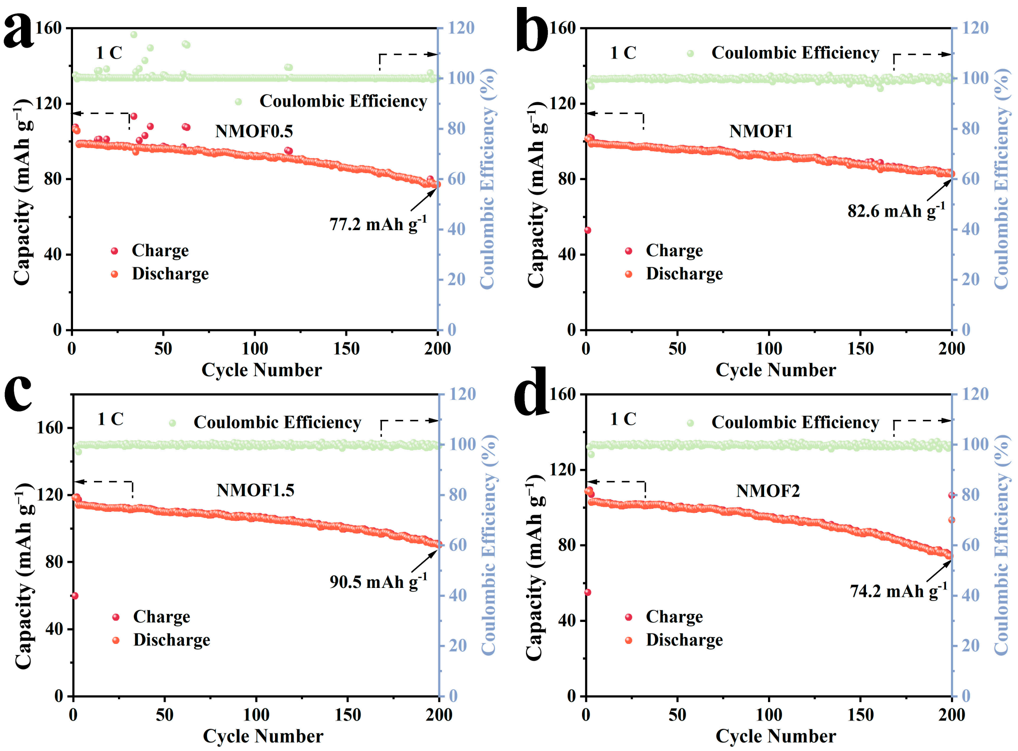

Figure S8. Cycling performance of the NMOF cathodes at 1 C: (a) NMOF0.5, (b) NMOF1, (c) NMOF1.5, (d) NMOF2.

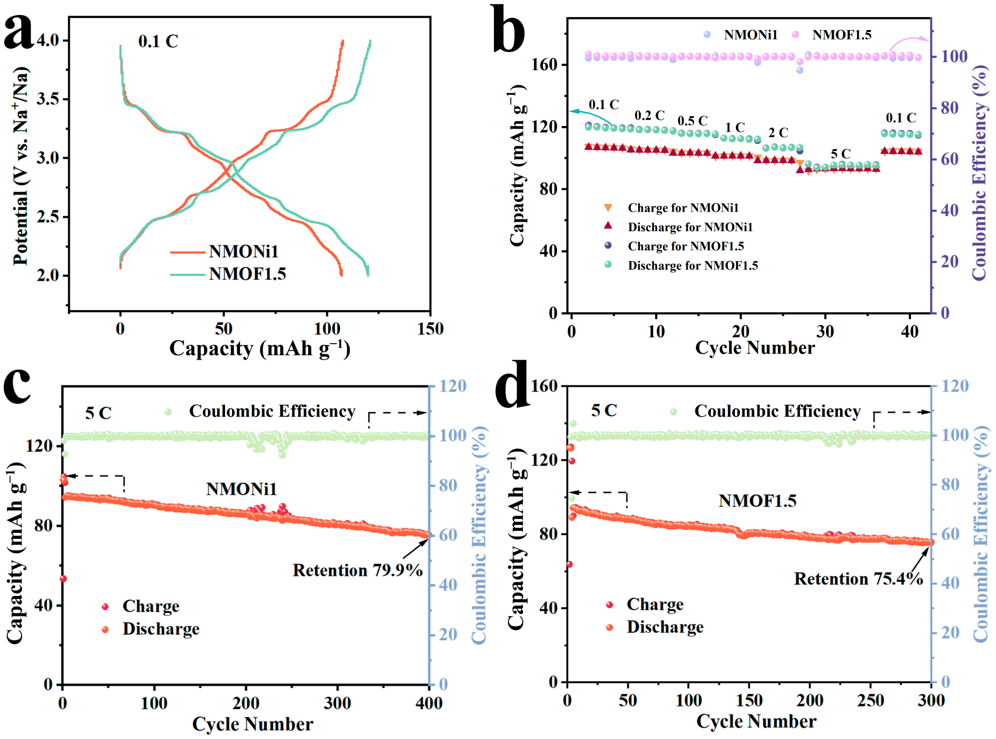

Figure S9. Electrochemical performance of NMONi1 and NMOF1.5 cathodes. (a) The galvanostatic charge-discharge curves for the second cycle at 0.1 C. (b) Rate capability. (c, d) Cycling performance at 5 C.

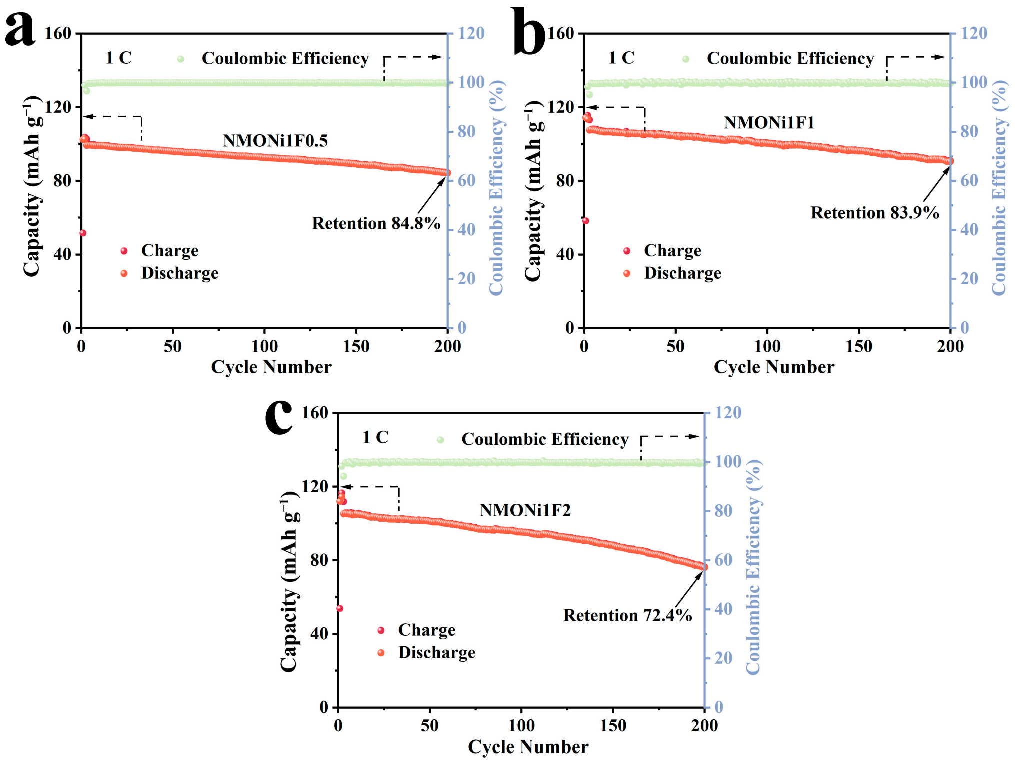

Figure S10. Cycling performance of the NMONiF cathodes at 1 C: (a) NMONi1F0.5, (b) NMONi1F1, (c) NMONi1F2.

.

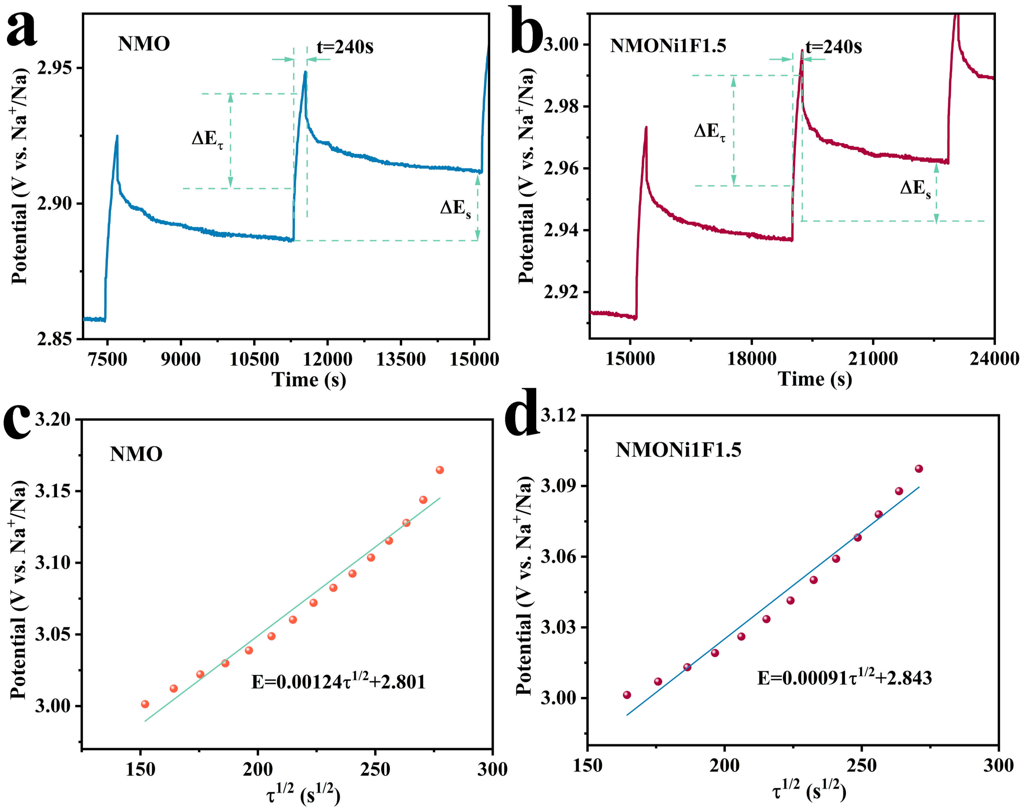

Figure S11. (a, b) Schematic diagram of a single-step titration of NMO and NMONi1F1.5 during GITT measurement. The linear relationship of (c) NMO and (d) NMONi1F1.5 between the potential and $\tau^{1/2}$ during the titration.

The Na^+ diffusion coefficient (D_{Na^+}) in the single-phase regions can be calculated by the following equation (S1):

$$D_{Na^+} = \frac{4}{\pi\tau}\left(\frac{m_B V_M}{M_B S}\right)^2 \left(\frac{\Delta E_S}{\Delta E_\tau}\right)^2 \tag{S1}$$

where m_B, V_M, and M_B denote active materials parameters including mass, molar volume, and molecular weight. S is the geometric area of the electrode. ΔE_S and ΔE_τ correspond to the difference between the steady-state voltage and the total voltage change during the constant current pulse time.

Table S1. Summary of fitted parameters in XRD Rietveld refinement.

Composition	*a* (Å)	*b*(Å)	*c*(Å)	V(Å^3)
NMO[a]	9.0810(3)	26.4513(4)	2.8229(9)	678.073
NMONi1F1.5	9.0913(5)	26.4620(5)	2.8282(2)	680.430

a: Rietveld refinement results of NMO cathode were obtained from our previous work (*Chem. Eng. J.* **2023**, 477, 146976) [22]

Table S2. The comparison of electrochemical performance between the previously reported Mn-based tunnel phase cathodes and this work.

Cathode	Potential window/V	Rate performance	Cycling performance	Reference
**$Na_{0.44}Mn_{0.99}Ni_{0.01}O_{1.985}F_{0.015}$**	**2.0–4.0**	**95.8 mAh g^{-1} at 5 C**	**89.4% after 400 cycles at 5 C**	***This work***
$Na_{0.44}Mn_{0.95}Mg_{0.05}O_2$	2.0–3.8	82 mAh g^{-1} at 5 C	72% after 800 cycles at 5 C	*Adv. Sci.* ***2021****, 8, 2004448 [24]*
$Na_{0.44}Mn_{0.9925}Co_{0.0075}O_2$	2.0–4.0	93 mAh g^{-1} at 10 C	85.2% after 500 cycles at 10 C	*ACS Appl. Mater. Interfaces* ***2020****, 12, 47548–47555 [25]*
$Na_{0.44}MnO_{1.93}F_{0.07}$	2.0–4.2	90 mAh g^{-1} at 5 C	79% after 400 cycles at 5 C	*J. Power Sources* ***2019****, 427, 129–137 [51]*
$Na_{0.44}Cu_{0.02}Mn_{0.98}O_{1.93}F_{0.07}$	2.0–4.1	79 mAh g^{-1} at 5 C	78.3% after 400 cycles at 3 C	*Energy Stor. Mater.* ***2024****, 65, 103161 [37]*
tt-$Na_{0.44}MnO_2$	1.7–4.0	96 mAh g^{-1} at 3 C	80% after 200 cycles at 2 C	*J. Mater. Chem. A,* ***2024****, 12, 25109–25116 [52]*
$Na_{0.61}[Mn_{0.27}Fe_{0.34}Ti_{0.39}]O_2$	2.6–4.2	43 mAh g^{-1} at 10 C	90% after 100 cycles at 2 C	*Adv. Energy Mater.* ***2015****, 5, 1501156 [53]*

$Na_{0.44}Mn_{0.98}Zr_{0.02}O_2$	2.0−3.8	100 mAh g^{-1} at 5 C	80% after 1000 cycles at 5 C	*ChemElectroChem* ***2020****, 7, 2545–2552 [54]*
$Na_{0.44}Mn_{0.99}Mo_{0.01}O_2$	2.0−4.0	103.8 mAh g^{-1} at 1 C	77% after 100 cycles at 1 C	*Compos. Part B* ***2024****, 284, 111664 [32]*
$Na_{0.44}[Mn_{0.994}Fe_{0.006}]O_2$	1.5−3.8	79.1 mAh g^{-1} at 3 C	65% after 200 cycles at 0.5 C	*J. Colloid Interf. Sci.* ***2024****, 661, 389–400 [27]*
$Na_{0.44}Mn_{0.98}Nb_{0.02}O_2$	1.5−4.0	81.5 mAh g^{-1} at 3 C	82.3% after 800 cycles at 5 C	*Adv. Funct. Mater.* **2024***, 34, 2404442 [30]*
